# Circulating acyl-CoA-binding protein/diazepam-binding inhibitor in gestational diabetes mellitus

**DOI:** 10.1186/s12958-023-01152-z

**Published:** 2023-10-23

**Authors:** Robin Schürfeld, Ekaterine Baratashvili, Marleen Würfel, Matthias Blüher, Michael Stumvoll, Anke Tönjes, Thomas Ebert

**Affiliations:** 1 Medical Department III - Endocrinology, University Hospital Leipzig, 04103 Nephrology, Rheumatology, Leipzig, Germany; 2 Helmholtz Institute for Metabolic, Obesity and Vascular Research (HI-MAG, Helmholtz Zentrum München at the University of Leipzig and University Hospital, Leipzig, Germany

**Keywords:** Acyl-CoA-binding protein, Adipokines, Diazepam binding inhibitor, Gestational Diabetes Mellitus, Insulin resistance, Pregnancy

## Abstract

**Background:**

Acyl-CoA-binding protein (ACBP)/diazepam-binding inhibitor has recently been characterized as an endocrine factor affecting energy balance and lipid metabolism. However, regulation of ACBP in women with gestational diabetes mellitus (GDM) during pregnancy, as well as postpartum, has not been investigated, so far.

**Methods:**

ACBP was quantified in 74 women with GDM and 74 healthy, gestational age-matched, pregnant controls using an enzyme-linked immunosorbent assay. Furthermore, ACBP was quantified post-partum in 82 women (i.e. 41 women with previous GDM vs. 41 previous control women). ACBP was related to measures of obesity, hypertension, glucose and lipid metabolism, renal function, and inflammation during pregnancy and postpartum.

**Results:**

During pregnancy, median [interquartile range] ACBP levels were not significantly different in women with GDM (40.9 [40.0] µg/l) compared to healthy, pregnant controls (29.1 [32.3] µg/l) (p = 0.215). ACBP serum concentrations increased from 30.3 [40.5] µg/l during pregnancy to 59.7 [33.2] µg/l after pregnancy in the entire cohort (p < 0.001). This observed elevation was consistent across both subgroups of women, those with prior GDM and those without. Multivariate analysis revealed that homeostasis model assessment of beta cell function (HOMA2-B) and creatinine positively and independently correlated with serum ACBP after pregnancy, while multivariate analysis during pregnancy showed no significant correlations.

**Conclusions:**

Circulating ACBP is not a marker of GDM status, but ACBP is decreased during pregnancy, irrespective of GDM status. Furthermore, ACBP is related to beta cell function and renal markers in women after pregnancy.

## Introduction and background

Gestational diabetes mellitus (GDM) is a metabolic dysfunction during pregnancy which enhances the risk of acute and chronic adverse maternal, fetal, and neonatal outcomes [1] with a continuously increasing risk in parallel with worsening maternal glycemia [2]. A pivotal element in the progression of GDM is the presence of insulin resistance [3]. Over the past few decades, multiple cytokines that contribute to the genesis of insulin resistance in both type 2 diabetes (T2D) and GDM have been identified [4–6]. Hence, circulating levels of the adipokine adiponectin are decreased in T2D and serve as an independent and unfavorable prognostic factor for GDM (as discussed in [5, 6]). Additionally, the metabolically adverse hepatokine fetuin B exhibits elevated levels in both T2D and GDM [7, 8]. Consequently, circulating cytokines potentially act as mediators for metabolically adverse impacts and actively contribute to the etiology of insulin resistance conditions, such as T2D and GDM.

Recently, acyl-CoA binding protein (ACBP), also referred to as diazepam binding inhibitor, has emerged as a potential modulator of food intake and lipid metabolism [9], and a relevant peripheral appetite-stimulating factor [10]. In more detail, intravenous administration of recombinant ACBP to mice enhances food intake, reduces glucose levels, activates lateral hypothalamic orexigenic neurons, and increases lipogenic gene expression, e.g. fatty acid synthase in hepatocytes and white adipocytes [9]. Antagonizing this mechanism by ACBP-neutralizing antibodies induces hyperglycemia, hypophagia, and weight loss [9]. In line with these data, a systemic knockout (KO) of the ACBP gene leads to diminished weight gain upon a high-fat diet in adult mice compared to wild type mice [11]. ACBP KO mice display a heightened susceptibility to fasting-induced weight loss compared to controls [9]. In human studies, ACBP has also demonstrated a positive correlation with BMI, providing further support for its role as an obesogenic factor [9, 12, 13]. Lately, we could establish markedly increased ACBP levels in kidney failure (KF) and furthermore confirm results of different studies on ACBP´s independent association with dyslipidemia [12–14]. In addition, Montegut et al. associated high plasma concentrations of ACBP with an elevated risk of future cardiovascular disease [13]. Taken together, ACBP serves as a novel anabolic factor with potential relevant metabolic function also during pregnancy and GDM. In contrast to different metabolic disease states in non-pregnant individuals, there are no studies investigating ACBP in women with GDM compared to healthy pregnant controls, as well as in a *post-partum* cohort, so far. Therefore, we have quantified serum levels of ACBP in 74 women with GDM during pregnancy as compared to 74 gestational age-matched, healthy pregnant controls. Furthermore, ACBP was quantified *post-partum* in 41 women with and without previous GDM at a follow-up time point, respectively. ACBP was correlated to measures of obesity, hypertension, indices of glucose metabolism/insulin resistance, lipid metabolism, renal function, and inflammation, both during and after pregnancy.

We hypothesize that ACBP is increased in women with GDM and is independently associated with markers of an impaired metabolic status.

## Methods

### Study participants

For this prospective cross-sectional study, we have analyzed data collected from a population of pregnant women with GDM as compared to healthy pregnant controls. Briefly, 148 pregnant women were recruited from the outpatient care unit of the Department of Endocrinology and Nephrology, University of Leipzig between 2006 and 2011 [7, 15, 16]. In all women, standardized questionnaires to assess past medical history and family history, anthropometric parameters, as well as a 75 g, 2 h oral glucose tolerance test (OGTT) were performed in the second trimester at a median gestational age of 201 days (interquartile range: 36 days). GDM diagnosis was confirmed using the following threshold plasma glucose levels: fasting ≥ 5.1 mmol/l; 1 h ≥ 10.0 mmol/l; 2 h ≥ 8.5 mmol/l according to the 2023 American Diabetes Association criteria [17]. Based on these criteria, 74 pregnant subjects were classified as GDM patients, while 74 gestational age-matched pregnant women with normal glucose tolerance served as controls. Body mass index (BMI) was determined as weight before gestation divided by squared height. Age and BMI before pregnancy did not differ between women with GDM and healthy pregnant controls.

To further investigate adaptations of ACBP after pregnancy, circulating ACBP was quantified in a postpartum group. For the current study, 82 individuals (41 previous controls, 41 previous GDM), were available. This subgroup of participants was selected from the initial study cohort of 148 pregnant women and underwent a follow-up examination, which took place with a median time of 1.574 days after delivery [interquartile range: 307 days].

The study was approved by the local Ethics Committee of the University of Leipzig, Germany, and all subjects gave written informed consent before taking part.

### Assays

All blood samples were obtained after an overnight fast. Blood specimens were immediately centrifuged and frozen at -80 °C until analyses were performed. Serum levels of ACBP (DBI Human enzyme-linked immunosorbent assay (ELISA) Kit, #KA0532, Abnova, Taipeh, Taiwan) were measured using an ELISA according to the manufacturer’s instructions. Fasting insulin (FI) was determined with a two-site chemiluminescent enzyme immunometric assay for the LIAISON automated analyzer (DiaSorin, Saluggia, Italy). All other parameters including lipids (i.e. high-density lipoprotein (HDL), low-density lipoprotein (LDL), total cholesterol, triglycerides (TG), and free fatty acids (FFA)), renal markers (i.e. creatinine), glycated hemoglobin A1c (HbA1c), glucose levels during the OGTT, and inflammation markers (i.e. high sensitivity C reactive protein (hsCRP)) were measured by standard laboratory methods in a certified laboratory using the Cobas Modular Analyzer Series (Roche, Basel, Switzerland). Using fasting glucose and FI, homeostasis model assessment of insulin resistance (HOMA2-IR) and beta cell function (HOMA2-B) were determined using the publicly available HOMA2 Calculator (https://www.rdm.ox.ac.uk/about/our-clinical-facilities-and-mrc-units/DTU/software/homa; accessed July 2023). eGFR was calculated as defined by the Chronic Kidney Disease Epidemiology Collaboration (CKD-EPI) formula [18].

### Statistical analysis

SPSS software version 29.0 (IBM, Armonk, NY) and GraphPad Prism 9 (GraphPad Software Inc., La Jolla, CA) were used in all statistical analyses. Group differences between women with GDM and healthy pregnant control women were assessed by non-parametric Mann–Whitney U test. Differences in circulating ACBP during pregnancy and postpartum were assessed by non-parametric Wilcoxon signed rank test. Univariate correlations were performed using non-parametric Spearman’s rank correlation method. In a second step, multivariate linear regression analysis was performed to identify independent relationships between ACBP and cardiometabolic covariates. In multivariate regression analysis, only parameters that correlated significantly with ACBP in univariate analysis were included. Prior to carrying out multivariate linear regression analysis, all non-normally distributed parameters were logarithmically transformed.

A p-value of < 0.05 was considered as statistically significant in all analyses.

## Results

### Baseline characteristics of the entire cohort (N = 148) during pregnancy

In the total sample comprising of 74 women with GDM and 74 healthy, pregnant controls, median [interquartile range] serum level of ACBP was 31.1 [39.7] µg/l. Clinical characteristics of the two subgroups (i.e. women with GDM vs. pregnant women without GDM) are shown in Table 1. Median ACBP levels were not significantly different in women with GDM (40.9 [40.0] µg/l) compared to healthy, pregnant controls (29.1 [32.3] µg/l) (p = 0.215) (Table 1). In contrast to ACBP, plasma glucose levels during OGTT, FI, HOMA2-IR, and FFA were significantly higher in women with GDM as compared to controls (p < 0.05) (Table 1). There was no significant difference in age, gestational age at blood sampling, gestational age at delivery, birth weight, as well as markers of obesity, hypertension, dyslipidemia, renal function, and inflammation (Table 1).

**Table 1:** Baseline characteristics of the study population during pregnancy

	Controls	GDM	p
N	74	74	
ACBP (µg/l)	29.1 (32.3)	40.9 (40.0)	0.215
Age (years)	28.9 (4.5)	31.0 (7.5)	0.087
Gestational age at blood sampling (days)	199 (40)	202 (33)	0.566
Gestational age at delivery (days)	275 (15)	273 (14)	0.311
Infant birth weight (g)	3360 (790)	3400 (805)	0.472
BMI (kg/m²)	22.4 (6.7)	24.5 (6.6)	0.117
SBP (mmHg)	125 (17)	120 (20)	0.336
DBP (mmHg)	75 (13)	73 (15)	0.348
HbA1c (%)	5.3 (0.6)	5.4 (0.6)	0.728
HbA1c (mmol/mol)	34.4 (6.6)	35.5 (6.6)	0.728
Glucose 0 h_(OGTT)_ (mmol/l)	4.3 (0.5)	4.5 (0.9)	< 0.001*
Glucose 1 h_(OGTT)_ (mmol/l)	7.5 (1.6)	10.1 (1.7)	< 0.001*
Glucose 2 h_(OGTT)_ (mmol/l)	6.4 (1.8)	8.7 (2.3)	< 0.001*
FI (pmol/l)	57.9 (38.4)	70.6 (66.7)	0.003*
HOMA2-IR	1.02 (0.68)	1.29 (1.17)	0.001*
HOMA2-B	135.3 (67.6)	129.5 (88.9)	0.991
Cholesterol (mmol/l)	6.3 (1.8)	6.7 (1.7)	0.198
HDL cholesterol (mmol/l)	1.9 (0.5)	1.8 (0.8)	0.417
LDL cholesterol (mmol/l)	3.7 (1.6)	4.1 (1.9)	0.568
TG (mmol/l)	2.0 (1.4)	2.1 (1.3)	0.451
FFA (mmol/l)	0.5 (0.3)	0.6 (0.3)	0.047*
Creatinine (µmol/l)	49.0 (11.0)	46.0 (11.3)	0.086
hsCRP (mg/l)	4.2 (4.3)	4.0 (6.1)	0.902

Baseline characteristics of the study population during pregnancy. ACBP, Acyl-CoA binding protein; BMI, Body mass index before pregnancy; SBP, Systolic blood pressure; DBP, Diastolic blood pressure; FI, Fasting insulin; HOMA2-IR, Homeostasis model assessment of insulin resistance; HOMA2-B, homeostasis model assessment of beta cell function; HDL, High density lipoprotein; LDL, Low density lipoprotein; TG, Triglycerides; FFA, Free fatty acids; eGFR, Estimated glomerular filtration rate; hsCRP, high sensitivity C-reactive protein. Values for median (interquartile range) are shown. Continuous parameters were analyzed by Mann–Whitney U test, * indicates p < 0.05.

### Univariate correlations and multivariate regression analysis of the entire cohort (N = 148) during pregnancy

ACBP positively correlated with FI, HOMA2-IR, HOMA2-B and FFA in pregnant women in univariate correlation analyses (all p < 0.05) (Table 2). Multivariate regression analysis for ACBP (dependent variable) and age, HOMA2-IR, FFA, and GDM status (independent variables) did not reach overall significance (overall significance level for the multivariate model: p = 0.063), therefore not allowing independent associations of single metabolic markers witch ACBP (Table 2).

**Table 2:** Univariate correlations and multivariate regression analysis of the study population during pregnancy

	Univariate correlation analyses		Multivariate linear regression analysis	
	r	p	β	p
Age (years)	-0.054	0.516	-0.073	n.s.
GDM status			0.029	n.s.
Gestational age at blood sampling (days)	-0.089	0.284		
Gestational age at delivery (days)	-0.062	0.473		
Infant birth weight (g)	0.005	0.950		
BMI (kg/m²)	0.126	0.132		
SBP (mmHg)	0.165	0.051		
DBP (mmHg)	0.048	0.573		
HbA1c (%)	0.020	0.816		
HbA1c (mmol/mol)	0.020	0.816		
Glucose 0 h_(OGTT)_ (mmol/l)	-0.025	0.763		
Glucose 1 h_(OGTT)_ (mmol/l)	0.031	0.716		
Glucose 2 h_(OGTT)_ (mmol/l)	-0.028	0.744		
FI (pmol/l)	0.172	0.037*		
HOMA2-IR	0.177	0.031*	0.127	n.s.
HOMA2-B	0.164	0.047*		
Cholesterol (mmol/l)	0.087	0.296		
HDL cholesterol (mmol/l)	-0.029	0.727		
LDL cholesterol (mmol/l)	0.030	0.719		
TG (mmol/l)	0.029	0.729		
FFA (mmol/l)	0.224	0.006*	0.165	n.s.
Creatinine (µmol/l)	0.063	0.445		
hsCRP (mg/l)	0.047	0.572		

Univariate correlation analyses and multivariate linear regression analysis of ACBP with various parameters in the study population during pregnancy. Non-parametric Spearman’s rank correlation method was used to assess univariate relationships between ACBP and indicated markers. Multivariate regression analysis was calculated for ACBP (lg, dependent variable) and adjusted for age (lg), GDM status, HOMA2-IR (lg), and FFA (lg), but the model did not reach overall significance. Therefore, single p values for these metabolic markers are not given (n.s. = not significant). r- and p-values, as well as standardized β-coefficients, are given. Abbreviations are indicated in Table 1.

### Baseline characteristics and changes of ACBP in the postpartum follow-up cohort (N = 82)

**Fig. 1:**
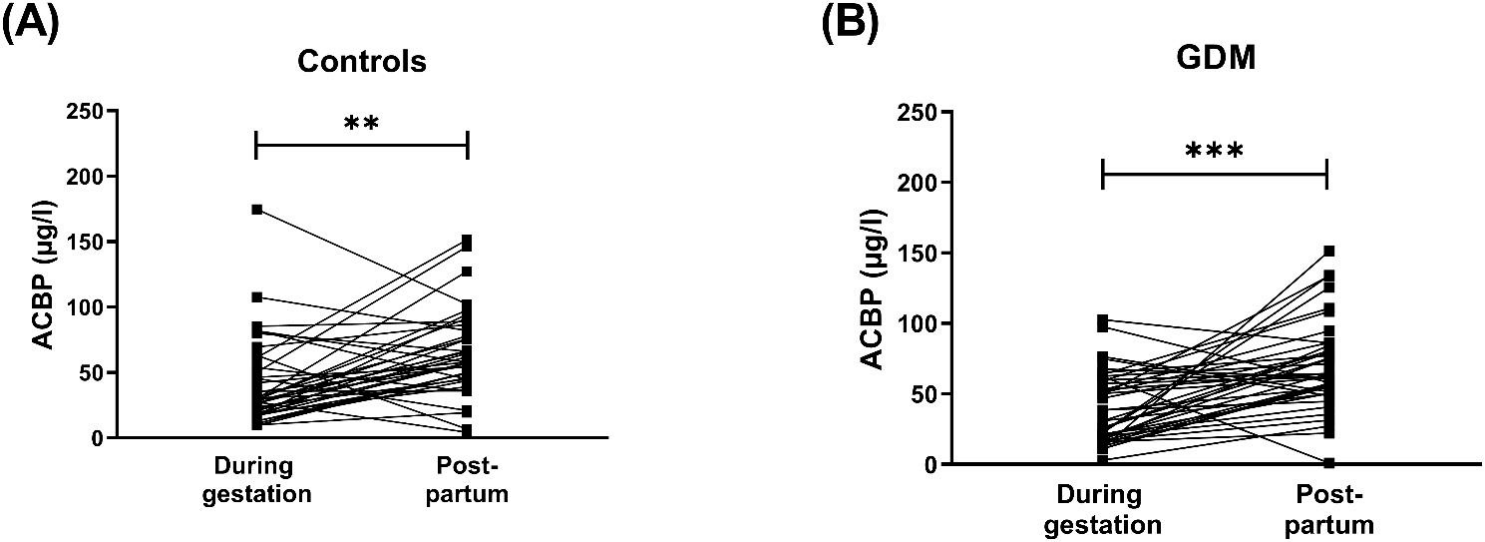
Serum levels of ACBP in pre- and postpartum samples in (**A**) healthy, pregnant women (controls), as well as in (**B**) women with gestational diabetes mellitus (GDM). N = 74 during gestation and N = 41 at follow-up post-partum time-point for both groups. ** indicates p < 0.01 and *** indicates p < 0.001 as assessed by Wilcoxon signed rank test

In total, about 82 (previous healthy controls: N = 41, previous GDM: N = 41) women were available for the follow-up study. Median ACBP levels significantly increased from 30.3 [40.5] µg/l during pregnancy to 59.7 [33.2] µg/l at the post-partum time point in the entire cohort (p < 0.001). Analyzing both subgroups separately, ACBP was significantly higher after pregnancy compared to the prepartum time point for both groups, i.e. women with GDM (30.8 [41.2] µg/l during pregnancy, 62.0 [31.7] µg/l after pregnancy, p < 0.001) and without GDM (29.3 [38.6] µg/l during pregnancy, 57.4 [35.7] µg/l after pregnancy, p = 0.003), respectively (Fig. 1).

Further clinical und biochemical characteristics for women with prior GDM compared to the control group are shown in Table 3. Postpartum ACBP concentrations did not differ between both groups (GDM: 62.0 [31.7] µg/l, control: 57.4 [35.7] µg/l, p = 0.288, Table 3). Other metabolic parameters did also not differ between both groups, e.g. markers of lipid metabolism like cholesterol (Table 3). In contrast, time after delivery was significantly increased in the control group (Table 3).

**Table 3:** Baseline characteristics of the postpartum follow-up cohort

	Controls	GDM	p
N	41	41	
ACBP (µg/l)	57.4 (35.7)	62.0 (31.7)	0.288
Age (years)	34.2 (9.0)	35.8 (7.5)	0.885
Time after delivery (days)	1610 (221)	1455 (728)	0.045*
Waist-to-hip Ratio	0.88 (0.08)	0.85 (0.12)	0.182
BMI (kg/m²)	23.2 (5.0)	25.2 (5.3)	0.444
SBP (mmHg)	115 (19)	120 (15)	0.138
DBP (mmHg)	75 (19)	80 (15)	0.068
HbA1c (%)	5.0 (0.22)	5.0 (0.35)	0.283
HbA1c (mmol/mol)	30.8 (2.4)	31.2 (3.9)	0.283
Fasting Glucose (mmol/l)	4.8 (0.4)	4.9 (0.6)	0.071
FI (pmol/l)	55.2 (65.3)	64.5 (42.3)	0.688
HOMA2-IR	1.0 (1.2)	1.2 (0.8)	0.674
HOMA2-B	107.4 (82.9)	97.0 (54.8)	0.674
Cholesterol (mmol/l)	5.0 (1.0)	5.1 (1.3)	0.086
HDL cholesterol (mmol/l)	1.6 (0.4)	1.6 (0.6)	0.824
LDL cholesterol (mmol/l)	3.0 (0.9)	3.2 (1.2)	0.155
TG (mmol/l)	0.9 (0.6)	1.1 (0.7)	0.180
FFA (mmol/l)	0.4 (0.3)	0.6 (0.4)	0.171
Creatinine (µmol/l)	67.5 (15.5)	65 (14.5)	0.762
eGFR (ml/min/1.73m^2^)	98.5 (22.7)	105.2 (22.2)	0.392
hsCRP (mg/l)	1.6 (3.6)	1.1 (5.2)	0.571

Baseline characteristics of the postpartum follow-up cohort. Abbreviations are indicated in Table 1. Values for median (interquartile range) are shown. Continuous parameters were analyzed by Mann–Whitney U test, * indicates p < 0.05.

### Univariate correlations and multivariate regression analysis in the postpartum follow-up cohort (N = 82)

In the entire postpartum cohort (N = 82), ACBP negatively correlated with time after delivery (p < 0.001) and FG (p < 0.05), while HOMA2-B positively correlated with ACBP (p < 0.05). Furthermore, ACBP was positively related to serum creatinine (p < 0.05) (Table 4). To verify independent associations, multiple linear regression analysis was performed. Here, HOMA2-B and creatinine remained independent, positive predictors of circulating ACBP levels after adjustment for age, GDM status, time after delivery, HOMA2-B, and creatinine.

**Table 4:** Univariate correlations and multivariate regression analysis of the of the postpartum follow-up cohort

	Univariate correlation analyses		Multivariate linear regression analysis	
	r	p	β	p
Age (years)	-0.008	0.940	0.058	0.606
GDM status			0.115	0.316
Time after delivery (days)	-0.424	< 0.001*	0.050	0.682
Waist-to-hip Ratio	-0.108	0.341		
BMI (kg/m²)	0.063	0.583		
SBP (mmHg)	0.030	0.795		
DBP (mmHg)	-0.034	0.766		
HbA1c (%)	0.130	0.248		
HbA1c (mmol/mol)	0.130	0.248		
Fasting Glucose (mmol/l)	-0.230	0.039*		
FI (pmol/l)	0.165	0.142		
HOMA2-IR	0.158	0.158		
HOMA2-B	0.243	0.029*	**0.339**	0.002*
Cholesterol (mmol/l)	0.163	0.145		
HDL cholesterol (mmol/l)	-0.079	0.486		
LDL cholesterol (mmol/l)	0.212	0.058		
TG (mmol/l)	0.063	0.577		
FFA (mmol/l)	0.138	0.219		
Creatinine (µmol/l)	0.268	0.015*	**0.288**	0.008*
eGFR (ml/min/1.73m^2^)	-0.256	0.022*		
hsCRP (mg/l)	-0.008	0.943		

Univariate correlation analyses and multivariate linear regression analysis of ACBP with various parameters in the follow-up cohort. Non-parametric Spearman’s rank correlation method was used to assess univariate relationships between ACBP and indicated markers. Multivariate regression analysis was calculated for ACBP (lg, dependent variable) adjusted for age (lg), GDM status, time after delivery (lg), HOMA2-B (lg), and creatinine (lg). r- and p-values, as well as standardized β-coefficients and p-values, are given. Abbreviations are indicated in Table 1.

## Discussion

In the present study, we demonstrated that circulating serum ACBP is not significantly different in women with GDM compared to healthy pregnant controls. Furthermore, ACBP levels did not differ between these groups at a postpartum follow-up time point. However, our data show that ACBP is significantly decreased during pregnancy by ~ 50%, irrespective of GDM status. Moreover, HOMA2-B and creatinine remain positive and independent predictors of circulating ACBP after pregnancy.

Previous studies indicate that ACBP is increased in overweight and obese individuals [9, 12, 13]. Furthermore, rs2084202, a specific SNP in the promoter region of the splice variant ACBP1c, has been associated with a decreased risk for T2D [19]. In contrast, another study did not find any difference in circulating ACBP concentrations in individuals with diabetes and prediabetes compared to controls with normal glucose tolerance [20]. Whereas detailed studies on cardiometabolic associations exist in non-pregnant women, data on ACBP regulation during and after pregnancy is lacking.

In our cohort, pregnancy status itself was associated with significantly diminished circulating concentrations of ACBP in both groups, irrespective of GDM status. The exact underlying mechanisms for this, remain unclear, so far. Hypothetically, pregnancy could alter the tissue expression and synthesis of ACBP [21], for instance through placental-secreted factors, which may lead to increased serum concentrations after delivery. Interestingly, a differential regulation of glucose homeostasis-related cytokines between pregnant compared to non-pregnant populations have also been shown for the metabolically active cytokines proneurotensin [22], preadipocyte factor 1 [23], or sclerostin [24]. Importantly, when comparing seven different cytokines in pregnant participants from this cohort (both GDM and non-GDM) to non-diabetic, age and BMI-matched non-pregnant women, most of the investigated seven cytokines can discriminate only pregnancy status, but not GDM status. Thus, it is tempting to speculate whether systemically altered ACBP levels due to the pregnancy status itself is the cause of the non-significant difference between GDM and non-GDM pregnant women.

Apart from this, HOMA2-B as a marker of beta cell function was positively associated with ACBP levels after pregnancy in the total cohort in multivariate correlation analyses, indicating increased activity of beta cells with increasing ACBP concentrations. Furthermore, in our cohort during pregnancy, insulin levels and insulin resistance, quantified by HOMA2-IR, positively correlated with raised ACBP concentrations in univariate analyses. In line with this, in non-pregnant human cohorts, ACBP has been associated with elevated insulin levels in obese individuals, while starved mice injected with anti-ACBP antibodies exhibited a decrease in plasma insulin [9]. Moreover, it is known, that ACBP is linked to an adverse, insulin-resistance favoring lipid profile with increased TG and decreased HDL cholesterol [12–14]. Hence, the pathophysiological connection between ACBP and insulin resistance may lead to enhanced insulin secretion of beta cells in order to overcome this insulin resistant state.

In addition, ACBP levels positively and independently correlate with creatinine concentrations in the post-partum cohort, suggesting increased ACBP levels with impaired renal function. This is in accordance with a recent study from us demonstrating significantly increased ACBP concentrations in patients with kidney failure and acute kidney dysfunction [14].

In further correlation analyses, ACBP and BMI in the cohort during pregnancy, as well as in the post-partum cohort, are not associated, which contrasts earlier studies [9, 12, 13]. However, our correlation analyses for BMI and ACBP during pregnancy in fact refer to the BMI value prior to pregnancy. Therefore, it would be interesting to investigate in future studies, whether ACBP levels longitudinally during pregnancy correlate with actual weight gain during pregnancy. Importantly, body weight gain during pregnancy is not only due to fat mass increase, whereas ACBP has been closely linked to biochemical pathways in adipose tissue in non-pregnant cohorts. Thus, associations of ACBP with fat mass (for instance measured by bioelectrical impedance analysis) during pregnancy might detect a link between ACBP and adipose tissue mass more comprehensively than pre-gestational BMI. In accordance with this hypothesis, anti-ACBP antibodies reduce absolute fat mass under a high-fat diet in mice, and periumbilical fat expresses high levels of *ACBP* mRNA that diminishes upon dietary intervention in patients with obesity [9].

Some limitations of our study need to be emphasized: First, our study has been performed in a prospective cross-sectional design, and, therefore, causality cannot be established. Moreover, follow up investigations were performed only in 82 women out of the 148 initial study participants resulting in a reduced power for longitudinal assessments. To confirm our results, future studies should aim to analyze a full female cohort during and after pregnancy, respectively. Furthermore, it would be interesting to study ACBP levels also at an early postpartum time point in order to validate a potential placental effect on circulating ACBP levels during pregnancy.

In conclusion, ACBP is not a diagnostic marker for GDM, but ACBP is decreased during pregnancy, irrespective of GDM status. Furthermore, ACBP is closely linked to beta cell function and renal markers in *post-partum* women.

## Data Availability

The datasets used and/or analyzed during the current study are available from the corresponding author on reasonable request.

## Abbreviations

ACBP acyl: CoA binding protein
BMI: Body mass index
FFA: Free fatty acids
GDM: Gestational diabetes mellitus
HbA1c: Glycated hemoglobin A1c
HDL: Cholesterol high-density lipoprotein cholesterol
LDL: Cholesterol low-density lipoprotein cholesterol
OGTT: Oral glucose tolerance test
eGFR: Estimated Glomerular Filtration Rate
CRP: C-reactive Protein
HOMA2-IR: Homeostasis model assessment of insulin resistance
HOMA2-B: Homeostasis model assessment of beta cell function
